# The Endophytic Fungus *Chaetomium cupreum* Regulates Expression of Genes Involved in the Tolerance to Metals and Plant Growth Promotion in *Eucalyptus globulus* Roots

**DOI:** 10.3390/microorganisms7110490

**Published:** 2019-10-26

**Authors:** Javier Ortiz, Javiera Soto, Alejandra Fuentes, Héctor Herrera, Claudio Meneses, César Arriagada

**Affiliations:** 1 Laboratorio de Biorremediación, Facultad de Ciencias Agropecuarias y Forestales, Universidad de La Frontera, Francisco Salazar 01145, Temuco, Chile; j.ortiz01@ufromail.cl (J.O.); javiera.psp@gmail.com (J.S.); alejandra.fuentes@ufrontera.cl (A.F.); hector.herrera@ufrontera.cl (H.H.); 2 Programa de Doctorado en Ciencias Mención Biología Celular y Molecular Aplicada, Universidad de La Frontera, Francisco Salazar 01145, Temuco, Chile; 3 Centro de Biotecnología Vegetal, Facultad de Ciencias Biológicas, Universidad Andrés Bello, Santiago, Chile; clamenes@gmail.com

**Keywords:** *Chaetomium cupreum*, endophyte, *Eucalyptus globulus* roots, metal stress, transcriptome

## Abstract

The endophytic strain *Chaetomium cupreum* isolated from metal-contaminated soil was inoculated in *Eucalyptus globulus* roots to identify genes involved in metal stress response and plant growth promotion. We analyzed the transcriptome of *E. globulus* roots inoculated with *C. cupreum*. De novo sequencing, assembly, and analysis were performed to identify molecular mechanisms involved in metal stress tolerance and plant growth promotion. A total of 393,371,743 paired-end reads were assembled into 135,155 putative transcripts. It was found that 663 genes significantly changed their expression in the presence of treatment, of which 369 were up-regulated and 294 were down-regulated. We found differentially expressed genes (DEGs) encoding metal transporters, transcription factors, stress and defense response proteins, as well as DEGs involved in auxin biosynthesis and metabolism. Our results showed that the inoculation of *C. cupreum* enhanced tolerance to metals and growth promotion on *E. globulus*. This study provides new information to understand molecular mechanisms involved in plant–microbe interactions under metals stress.

## 1. Introduction

In Chile, copper (Cu) mining has generated an over-accumulation of metals in areas surrounding Cu smelters, and Puchuncaví Valley (Valparaíso region, Chile) is a recognized place by the high environmental pollution originated mainly for Cu smelting. The toxicity of metals can generate losses of vegetal diversity and functionality of species, leading to a change of the soil characteristics and difficulty to establish vegetation [1]. However, it is known that prolonged exposure to metals can generate a selection of resistant/tolerant plant populations [2]. *Eucalyptus* spp. has been reported as a metal-tolerant species and used for phytoremediation process due to it fast growth; high biomass production; wide adaptability; and accumulation of high amounts of metals such as Cu, Zn, Pb, and Cd [3]. 

Soil microorganisms play an important role in the restoration of environments affected by contamination of metals, promoting plant growth through different mechanisms such as indole acetic acid (IAA) production, siderophores, organic acids, biosurfactants, and phosphate solubilization [4]. Among these, endophytic fungi may confer physiological and ecological benefits to the host, including enhanced growth, protection from attack of pathogens, help establishment in degraded ecosystems, and tolerate different types of stress [5].

Previous reports have shown that endophytic fungi inoculation enhances plant growth and tolerance in hosts under metal stress [6]. For example, endophytic fungus *C. cupreum* enhances growth and tolerance to Cu in *E. globulus* plants cultivated in soils with high concentrations of metals and metalloids [7]. The inoculation of the strain *Penicillium funiculosum* LHL06 to *Glycine max* plants reduces metal accumulation and activates signaling of stress-response and antioxidant systems [8]. While the inoculation of *Gaeumannomyces cylindrosporus* increased root length and biomass under lead stress, moreover, it altered translocation and accumulation of lead in *Zea mays* plants [9].

The development of new sequencing technologies has contributed to characterizing molecular responses to abiotic stress in several plants [10,11]. Among them, RNA-sequencing (RNA-seq) has been successfully applied in transcriptomic studies providing information about levels of transcripts and changes in gene expression even in non-model species [12,13,14]

Recent studies have delved into the molecular mechanisms involved in the tolerance of plants to metal stress. However, only a few transcriptomic studies have focused on understanding how the inoculation of endophytic microorganisms in plants can contribute to the response to metal stress, considering that there are some microorganisms that provide beneficial effects for the growth and productivity of plants through different molecular mechanisms [15].

On the other hand, several studies have described mechanisms involved in Cu tolerance in plants, including: i) a reduction of the uptake of Cu by the plant or ii) an increase of the efflux of Cu [16], iii) sequestration and compartmentalization [17], iv) extracellular precipitation [18], and v) high regulation of antioxidant defense systems [19]. Recently, Wang, et al. [12] described genes involved in Cu tolerance in *Paeonia ostii* finding that genes related to Cu transporters, plant hormone, signal transduction, transcription factors, and antioxidant systems play an important role in Cu stress response.

The understanding of plant–microbe interactions at the molecular level is an important aspect for the development of new phytoremediation alternatives based on the use of native microorganisms adapted to metal stress.

In the present research, we analyzed the transcriptomic response of *E. globulus* inoculated with *C. cupreum* growing in a multi-contaminated soil with metals. Analysis were carried out with de novo assembly of the transcriptomics of *E. globulus* roots tissues. The aim of this study was to identify genes involved in plant growth promotion and metal stress response, which provide important molecular information to understand plant–microbe–metal interactions and contribute to new strategies that can be used in phytoremediation processes.

## 2. Materials and Methods 

### 2.1. Plant Material and Experiment Design

Clonally propagated *E. globulus* seeds were acquired from a commercial nursery (Semillas Imperial, Los Ángeles, Chile). Seeds were germinated in vermiculite in a plant growth chamber at room temperature. After 4 weeks, uniform plants were selected and transplanted to 300 cc plastic pots with a mixture of Puchuncaví Valley soil: vermiculite (1:1 v/v) as a substrate. The soil is classified as an Entisol (Chilicauquén series) and has a pH_w_ of 5.54 and Cu, Zn, Pb, As and Cd content (in mg Kg^−1^) of 385, 183, 135, 52 and 1.1 respectively. Pots were randomly divided into two groups, without microbial inoculation as control while the other group was inoculated with fungal strain *C. cupreum* according to Almonacid, et al. [20], where one slant of active mycelia was diluted in 40 mL of sterile distilled water then homogenized and vigorously agitated (10 mL of this suspension were inoculated, equivalent to 70 mg of dry mycelium). Plants were grown in a greenhouse with supplementary light provided by incandescent cool white lamps (400 umol m^−2^ s^−1^, 400–700 nm) (Sylvania^®^, Wilmigton, MA) with a 16/8 h day/night cycle at 24/16 °C and 50% relative humidity. After 90 days post-inoculation (dpi), all plants were harvested and five biological samples were dried in an air-forced oven at 70°C for 48 h and then were weighted to determine the biomass production. Entire roots systems were collected from five biological replicates of the control and inoculated plants. All these samples were rinsed thoroughly with distilled water, immediately frozen in liquid nitrogen and stored at −80°C until RNA extraction.

### 2.2. Fungus Detection in Roots by Scanning Electron Microscopy (SEM)

For fungus visualization, six root segments collected at the end of the experiment (90 dpi) from non-inoculated and inoculated plants were cut into 1-cm pieces. Segments were obtained from the upper part (near to stem) and lower part (lateral roots). Samples were observed by SEM (Hitachi SU 3500, Japan).

### 2.3. RNA Extraction, Sequencing and Illumina Reads Processing

Total RNA was extracted from 70 mg of root tissue collected at the end of the experiment (90 dpi) using Spectrum^™^ Plant Total RNA kit (Sigma-Aldrich, Germany) following the manufacturer’s instructions. The yield and quality of the RNA isolation samples was measured using a Qubit^®^ 2.0 Fluorometer (Life Technolology, Carlsbad, CA), and Fragment Analyzer^™^ Automated CE System (Analytical Advanced Technologies, Ames, IA). To obtain good coverage of the *E. globulus* transcriptome, equal quantities of individual RNAs from root tissues of five biological replicate plants were used for library construction. Complementary DNA (cDNA) libraries were constructed using the TruSeq RNA Sample Preparation kit v2 (Illumina^®^, San Diego, CA) following the Illumina manufacturer’s instructions and subsequently sent for sequencing to Macrogen Inc (Seoul, Korea). A total of 10 samples were sequenced in a single lane of an Illumina HiSeq 4000 platform (Illumina) in paired-end mode for 101 cycles. The resulting FASTQ files containing Illumina raw sequences were analyzed and trimmed using NGSQC Toolkit v2.3 [21], removing adaptors and low-quality reads, based on their Q-score composition, removing all reads with a content of Q>30 lower than 70% of bases.

### 2.4. De novo Transcriptome Assembly

Due to the endophytic nature of *C. cupreum*, high-quality reads were aligned with *Eucalyptus grandis* genome using BLASTN. Matching reads were used to construct a de novo assembly using Trinity software v2.8.3 [22]. Transcriptome was assembled on an Amazon Web Service Linux instance m4.16xlarge and downstream analysis were carried out at Centro de Modelación y Computación Científica (CMCC, Universidad de La Frontera, Chile). Refinement of transcriptome was carried out mapping reads to assembled transcripts, and relative abundance in FPKM value (Fragments per kilobase per transcript per million mapped reads) was calculated with RSEM v1.2.26 [23]. Poorly supported transcripts were removed, keeping all transcripts with a relative abundance of at least 1 FPKM for downstream analysis. Highly similar and redundant transcripts were clustered using CD-HIT-EST with a threshold of 95% [24]. In order to validate the integrity of this de novo assembly, a comparison between *E. globulus* assembled transcriptome was carry out by BUSCO (Benchmarking Universal Single-Copy Orthologs) against OrthodBv9 database (embryophyte), to identify highly conserved orthologous genes [25].

### 2.5. Functional Annotation of the Transcriptome

The resulting transcripts were aligned into the SwissProt database using BLAST+ with an e-value filter of 1-e^−10^ as threshold, and evaluated in hidden Markov profiles to identify any family membership and conserved domains in PFAM-A database [26]. Functional annotation and Gene Ontology (GO) terms classification (Cellular component, Biological process and Molecular function and) were performed with PANTHER system [27], using as input gene lists obtained from blast top hit using reference proteomes collection from EMBL as database.

### 2.6. Differential Expression Analysis

The relative abundance was calculated using RSEM through align_and_estimate_abundance.pl script and the resulting abundance for each sample were merged in a matrix and analyzed with run_DE_analysis.pl script, which involves the Bioconductor package DESeq2 in R statistical environment [28]; both scripts were contained in the Trinity package. To judge the significance of gene expression, a False Discovery Rate value (FDR) lower than 0.05 and a minimum fold change (FC) of 2 were set as thresholds. Main DEGs related to metal response and plant growth promotion were analyzed through a heat map using log10 ratio values of expression levels. 

## 3. Results

### 3.1. Plant Biomass and Fungus Detection in Roots

**Figure 1:**
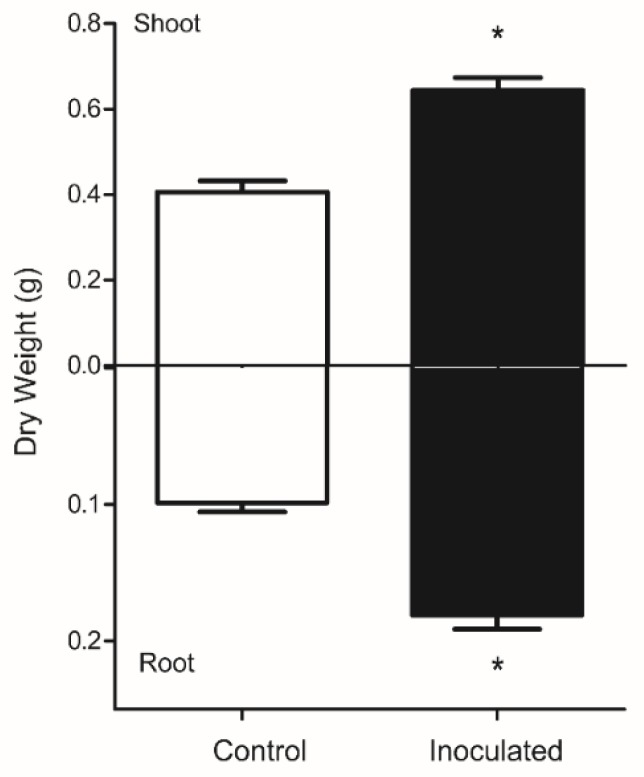
Effect of inoculation of endophytic strain *Chaetomium cupreum* on shoot and root dry weight of *Eucalyptus globulus* (90 days post-inoculation). Values are expressed as means ± standard error. Statistical significance was evaluated with *t*-student test *(*p* ≤ 0.05).

**Figure 2:**
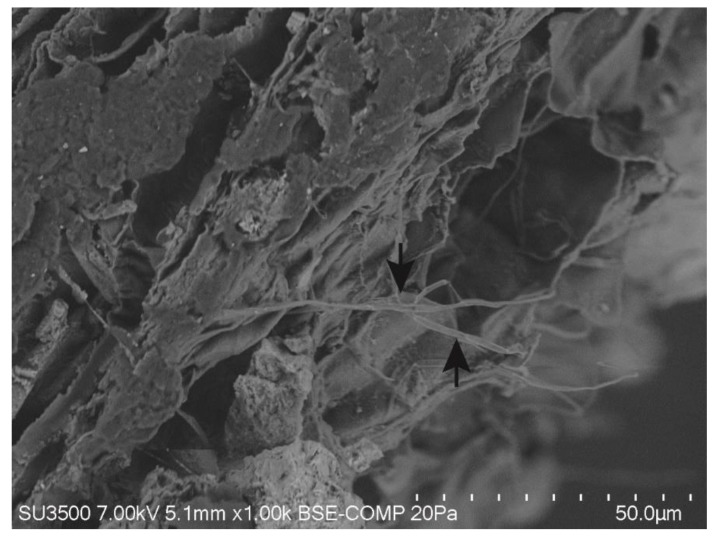
Detection of *Chaetomium cupreum* in roots of inoculated plants of *Eucalyptus globulus* (90 days post inoculation) observed under scanning electron microscopy. Black arrows show fungal hyphae.

After 90 dpi, inoculated plants showed significantly higher dry biomass as compared to the control, and increased 37% in shoots and 45% in roots in relation to non-inoculated plants (Figure 1). According to SEM images, no structures were observed in the roots of non-inoculated plants of *E. globulus* after harvest, while in all plants inoculated, fungal hyphae were observed (Figure 2).

### 3.2. Sequence Data, de novo Transcriptome Assembly and Annotation

Ten libraries, which include five control samples and five samples inoculated with *C. cupreum* (90 dpi), with a total of 460.158.421 paired-end reads, were obtained. After sequence trimming for adapter and filtering low quality reads, it resulted in 393.371.743 high quality reads (Table 1). De novo transcriptome assembly after trimming, where transcripts with an estimated abundance lower than 1 FPKM and highly similar or redundant transcripts with a sequence similarity higher than 95% were removed, resulted in 135.155 transcripts. Transcriptome statistics such as N50 and average length values can be observed in Table 1.

Transcripts of the final assembly were aligned to the Swissprot database; the homology search presented results for 37.667 sequences, corresponding to 27.9% of the total. De novo transcriptome was compared against BUSCO database, which contains information about highly conserved orthologous genes. Of the 1440 BUSCO genes, 1133 complete (78.7%), 108 fragmented (7.5%), and 199 missing genes (13.8%) were found in our assembly (Table 2).

### 3.3. Differential Expression Analysis

**Figure 3:**
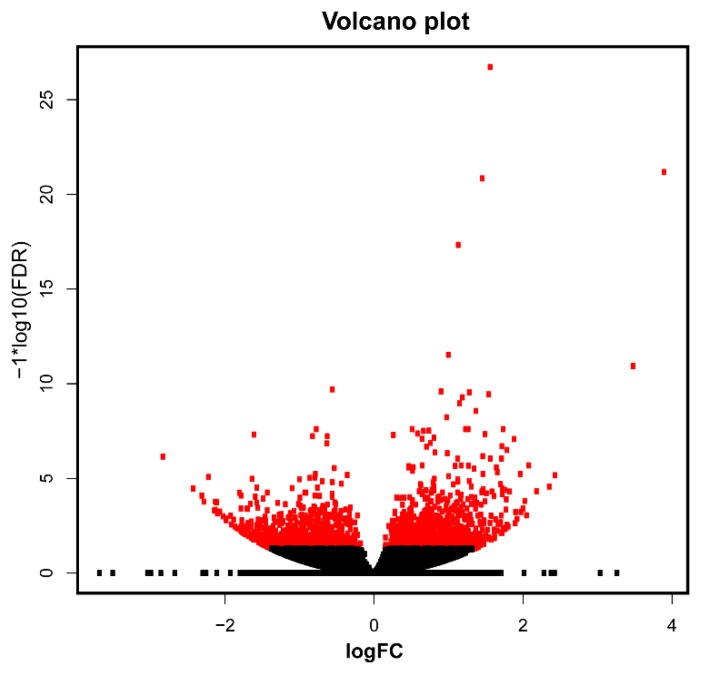
Volcano plot of differentially expressed genes between control (without microbial inoculation) and treatment (with microbial inoculation) conditions in *Eucalyptus globulus* roots transcriptome. In red, differentially expressed genes; in black, genes without variation in expression.

**Figure 4:**
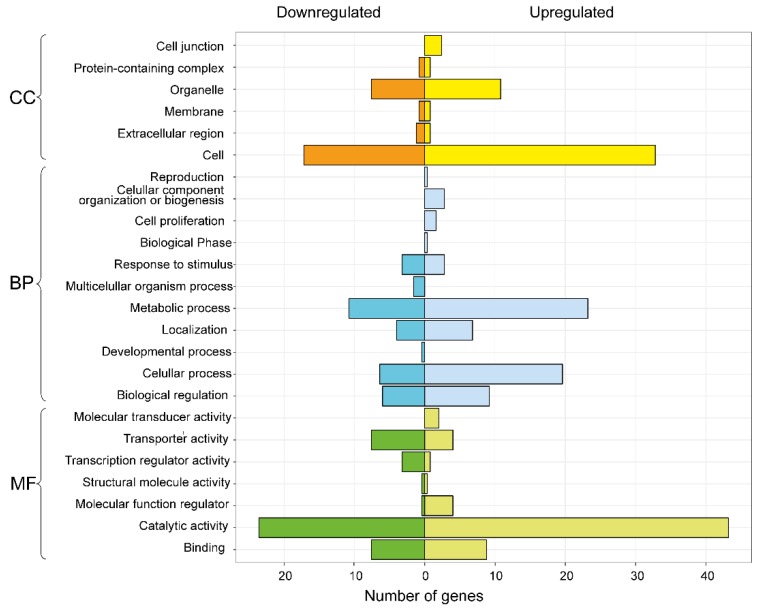
Gene ontology distribution of *Eucalyptus globulus* transcripts according to level 1 categories: GO, Cellular Component (CC), Biological Process (BP) and Molecular Function (MF). Genes are differentially expressed in treatment compared to control condition.

**Figure 5:**
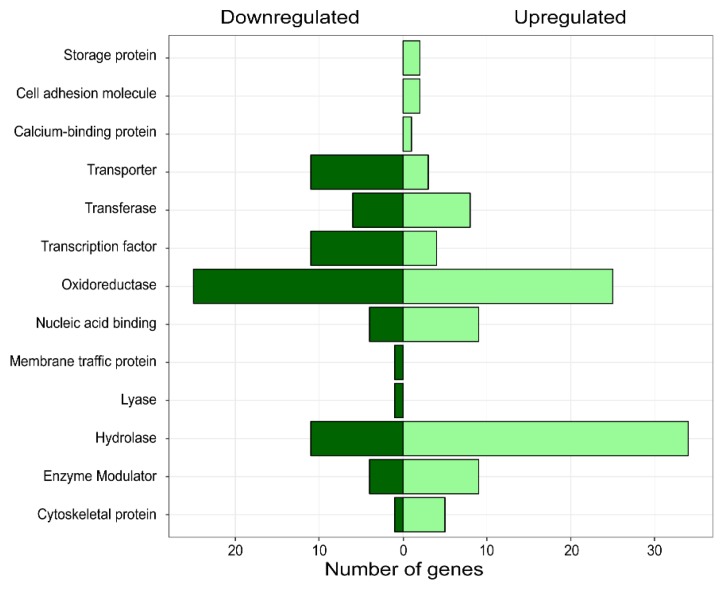
Distribution of differentially expressed genes according PHANTER classification system. The bars show the number of genes in each protein functional class. Genes are differentially expressed in treatment compared to control condition.

Differentially expressed genes (DEGs) were estimated using a fold change ≥ 2 and a FDR < 0.05 as cut-off between control and treatment conditions. In total, we found 709 DEGs (Figure 3), of which 403 were up-regulated and 306 were down-regulated in response to *C. cupreum* inoculation. However, those genes that did not match in annotation with Blast hit were removed and discarded. Therefore, we obtained 663 DEGs of which 369 were up-regulated and 294 down-regulated under *C. cupreum* inoculation. From DEGs, we investigated their functions carry out gene ontology analysis and classified them into three major GO terms (Figure 4). Between the annotated transcripts, cell and organelle had the two greatest number of transcripts in cellular component terms. For the biological process term, metabolic process, cellular process, and biological regulation had the most transcripts. Within molecular function, most transcripts showed catalytic activity, binding and transporter activity. According to the PHANTER classification system, we observed that the functional class transport and transcription factor were down-regulated in treatment compared to control, where a greater number of transcripts was presented (Figure 5).

Among identified DEGs, we explore those involved in response to metal stress, metal transport and plant growth promotion (Table 3). In the inoculated condition, we found down-regulated genes that play an important role against metal stress, among them: *metallothionein-like protein 1*, *peroxidase 10 precursor* and *heavy metal-associated isoprenylated plant protein 28* included in GO terms: metal ion binding (GO:0046872), response to oxidative stress (GO:0006979) and metal ion transport (GO:0030001), respectively. Other down-regulated genes were related to metals transport, *Natural resistance-associated macrophage protein 1* (*Nramp1*), *Nramp3*, *Nramp5*, *Nramp 6*, *Metal tolerance protein 4* (*MTP4*), and *Putative Multidrug Resistance Protein* (*MRP*) among others. Also, genes implicated in nutrient transport were down-regulated, including *high affinity nitrate transporter 2.5* (Nitrate transport, GO:0015706), *Inorganic phosphate transporter 1-1* (Phosphate ion transmembrane transporter activity, GO:0015114), *Potassium transporter 5* (Potassium ion transport, GO:0006813), *Sodium transporter HKT1* (Response to osmotic stress, GO: 0006970; Sodium transporter, GO:0006814), *Magnesium transporter MRS 2-3* (Magnesium ion transport, GO:0015693), and *Ammonium transporter 1 member 1* (Ammonium transmembrane transport, GO:0072488). However, *Sugar transporter ERD6-like 6*, *aquaporin TIP 2-1* and *probable aquaporin PIP2-2* were up-regulated.

In addition, genes related to other abiotic and biotic stress were identified; transcription factors *MYB 102*, *MYB 74* and *probable WRKY transcription factor 72* were down-regulated. While *MYB 86*, *Snakin-2* and *Disease resistance protein RPP4* were up-regulated. 

Several up-regulated genes involved in plant growth promotion via auxin production were identified, including: Auxin-induced protein 22A, auxin-induced protein 22D, auxin-induced protein AUX22, auxin-responsive protein IAA3, auxin-responsive protein IAA4, auxin efflux carrier component 2, auxin-induced in root cultures protein 12 precursor, auxin-responsive protein SAUR50, auxin-responsive protein SAUR78, and índole-3-acetic acid-amino synthetase GH3.17, which were mainly included in GO terms: Auxin-activated signaling pathway (GO:0009734), response to auxin (GO:0009733), positive regulation of cell growth (GO:0030307), auxin homeostasis (GO:0010252) and root development (GO:0048364). 

**Figure 6:**
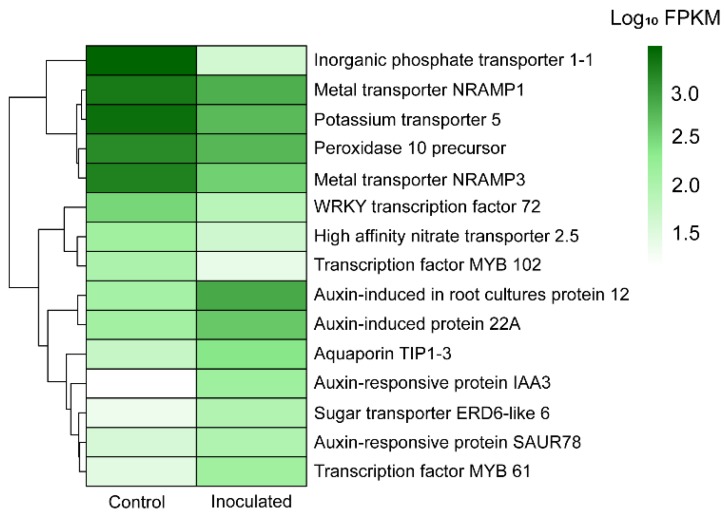
Heat map showing differential expression of the most important up/downregulated genes between control (without microbial inoculation) and inoculated treatment (with fungal inoculation) conditions in *Eucalyptus globulus* roots transcriptome.

Expression patterns between control and treatment conditions of the most important DEGs related to metal response and plant growth promotion are shown in Figure 6.

## 4. Discussion

Within abiotic stress types, metal toxicity is one of the factors that causes serious deleterious effects in plants. Nevertheless, the alleviation of heavy metal toxicity by endophytic fungi could be an efficient strategy to enhance heavy metal tolerance in plants [9]. In this study, colonization of *C. cupreum* was detected at the end of the experiment in samples of lateral roots and near to the stem of plants, which suggests that inoculation of *C. cupreum* is persistent over time. Endophytic fungi establish a chemical communication with the host through sugars, fatty acids, amino acids, polysaccharides, flavonoids, among others [29], and enters the plant through degradation of the cell wall or by fissure on roots [30]. Once inside the plant, the endophytic fungi can produce different chemical compounds with a beneficial effect on the performance of plants under heavy metal stress.

Exposure to metals causes the formation of reactive oxygen species (ROS) in plants, which leads to an imbalance in redox homeostasis [16]. Plants can counteract these negative effects by intracellular mechanisms such as the action of metal chelating peptides (metallothioneins and phytochelatins) and by activating antioxidant mechanisms. In our study, it was observed that inoculation of *C. cupreum* caused a down-regulation of genes involved in the detoxification of metals including, *metallothionein-like protein 1* that acts by sequestering metals through thiol groups of their cysteine residues, whose distribution influences the capacity of union and sequestration of metals to maintain homeostasis [31]. The metallochaperone *heavy metal-associated isoprenylated plant protein 28* (*HIPP28*), that acts against the excess of metals, binding them through their cysteine residues and transporting them to intracellular compartments, helping with the detoxification of metals and maintaining homeostasis [32], and *peroxidase 10 precursor* acts decomposing hydrogen peroxide generated in response to oxidative stress in addition to participating in the oxidation of reducing toxins, lignin biosynthesis, suberization and auxin metabolism, were also down-regulated [33].

On the other hand, several genes associated with the transport of metals were also down-regulated, among them: *Nramp1*, *Nramp3*, *Nramp5*, *Nramp 6*, *MTP4* and *MRP*. *Nramp* genes play an important role in the uptake and translocation of a wide range of metal ions to the plant that include Cd, Zn, Fe, Cu and Mn, and it has been described that an up-regulation of these genes improves the accumulation of metals in *Arabidopsis thaliana* [34]. *MTP4* has been described as a divalent cation efflux transporter that acts in the cytoplasm, being essential for the maintenance of metal homeostasis [35] and *MRP* realizes a similar role transporting metal ions to the vacuole as a detoxification mechanism of plants [36]. 

This suggests that the inoculation of *C. cupreum* prevents the metals present in the soil from being translocated to the roots of the plants of *E. globulus*, a finding which agrees with previous works where *C. cupreum* inoculation contributes positively to decreasing indicators of stress such as lipid peroxidation level and proline content because metal ions were adsorbed in the cell wall of the fungus [7]. However, this response mechanism depends directly on the plant–microorganism interaction, and on the type of host plant, because other studies showed that other endophytic species such as *Mucor sp.* despite having a protective effect on *Arabidopsis arenosa* plants by accumulating a lower amount of metals compared to the non-inoculated control, promotes translocation of metals from roots to shoots, which is reflected in an up-regulation of genes associated with metal transport and distribution such as *HMA3*, *PCR2*, *ZIF1*, and *MTP1* [37]. Other transporters down-regulated were *high affinity nitrate transporter 2.5*, *inorganic phosphate transporter 1-1*, *potassium transporter 5*, *sodium transporter HKT1*, *magnesium transporter MRS2-3*, *ammonium transporter 1 member 1* and *high affinity sulfate transporter 1*, which are directly related to plant nutrition. According to this, an increase in plant growth is not being promoted by an improvement in nutrient uptake. Other studies under *C. cupreum* inoculation reported similar results attributing the improvement in plant growth to a probable action of auxins [38]. Conversely, recent reports have shown an improvement in the absorption of nutrients under colonization of endophyte fungi. For example, the endophyte fungus *Serendipita indica* improved absorption and assimilation of phosphorus and nitrogen in *Cunninghamia lanceolata* plants under phosphorus starvation [39]. Similarly, *S. indica* increased the expression of genes encoding nitrate reductase in *Arabidopsis thaliana* [40]. While the endophyte fungus *Mucor sp.* increased the expression of genes related to phosphorous homeostasis in *A. arenosa* plants that developed in mining tailings [37]. Metalliferous environments are characterized by scarce vegetation, acid soils severely eroded and show high concentrations of metals, in addition to having a scarce supply of water and nutrients, which directly affects the development of plants [1]. In our study, *sugar transporter ERD6-like 6*, *aquaporin TIP 2-1* and *probable aquaporin PIP2-2* were up-regulated; these genes have important functions at the physiological level of the plant including nutrition and growth, providing energy through hexose accumulation and facilitating water transport respectively, besides participating in the adaptation to several types of stress [41,42]. The storage of osmoprotectants such as sugars and amino acids allows to maintain the cellular turgor pressure necessary for cell expansion under stressful conditions [43]. Previous reports suggest that endophytic fungi can promote sugar accumulation in adverse conditions, which improves plant fitness, limiting water losses by decreasing the transpiration rate and through an osmotic adjustment [44]. This could explain the up-regulation of genes related to sugar and water transport as well as Transcription factor (TF) *MYB86* that regulates the stomatic aperture.

In addition, DEGs related to other types of stress were identified, including TF *MYB102* and *MYB74* (down-regulated), which were involved in the response to osmotic and saline stress respectively, and *MYB86* (up-regulated) in the regulation of stomatal movement. It is well known that TFs can modulate the expression of genes, allowing them to respond and adapt to different environmental stimuli. MYB proteins participate in several important physiological processes, including control of the cell cycle, regulation of metabolism, synthesis of hormones, and response to several types of biotic and abiotic stresses [45]. Other genes were directly involved in plant defense response, among them, *Probable WRKY transcription factor 72* (down-regulated), whose expression is induced in the presence of pathogens and also under saline and osmotic stress [46]. The role of *WRKY transcription factor 72* has been described to modulate the resistance against *Xanthomonas oryzae* pathovar oryzae in rice plants [47]. *Snakin-2* (up-regulated), a peptide with antimicrobial activity, which acts drilling membranes of the microbial cells of pathogens [48]. Wherewith, it is inferred that the inoculation of *C. cupreum* not only gives benefits to the plant in terms of protection against metal stress, but also plays an important role in regulating the expression of genes related to other types of biotic and abiotic stress. 

In our study, we found genes related to biosynthesis and metabolism of auxins, which are responsible for the division, elongation and differentiation of the plant cell, and therefore, are directly involved with the growth of plants. We observed that genes classified as early response to auxins were up-regulated, whose function is to regulate cellular responses to different levels of auxins present in the plant [49]. Thereby, once the presence of auxins inside the cell is detected, several processes are triggered that modulate the expression of auxin response genes where two main families of proteins are involved, *Auxin/Índole-3-acetic acid* (Aux/IAA) and *Auxin response factor* (ARF). When there are low auxin concentrations, the repressor proteins Aux/IAA (*Auxin- induced protein AUX22*, *Auxin- induced protein 22D*, *Auxin- induce protein 22A*, *Auxin- responsive protein IAA3* and *Auxin- responsive protein IAA4*) form complexes with ARF proteins which regulate the expression of auxin-responsive genes, preventing their actions as transcription factors; while, when the auxin concentration increases, it binds to other receptors (TIR1 / AFB) together with other proteins (ASK1, CUL1 and RBX) and forms a complex of ubiquitination that binds to the AUX / IAA repressor proteins and degrades them in the 26S proteasome, releasing the ARF proteins to activate or repress the transcription again [50]. Other overexpressed genes were *Indole-3-acetic acid amino synthetase GH3* which were involved in the synthesis of IAA conjugates, providing a mechanism for the plant to counteract the excess of auxin, *auxin efflux carrier component 2,* which transports this phytohormone between different cells and tissues of the plant. *Small auxin upregulated RNAs* (*SAURs*) are the largest family of genes for early response to auxins and are closely related to cell expansion and plant growth along with regular abiotic stress tolerance responses such as saline and drought [51]. *Auxin-induced roots cultures protein 12* (*AIR12*) was also overexpressed and has been described as an induced auxin involved in the development of lateral roots [52]. In previous studies, we observed that under the inoculation of fungal strain, plant growth is stimulated [7] what was reflected in lateral roots proliferation and biomass production respect to control, demonstrating the plant growth promoting effect of fungus *C. cupreum*. 

## 5. Conclusions

Transcriptome changes in *E. globulus* inoculated with *C. cupreum* resulted in the detection of several genes involved in stress response to heavy metals and plant growth promotion. The protective effect showed by the inoculation of *C. cupreum* against metal stress is mainly due to the repression of metal transporters in the plant, which added to the ability of *C. cupreum* to fix metal ions on its cell surface (biosorption), preventing the generation of a ROS imbalance that subsequently triggers the activation of metal chelation mechanisms and activates the antioxidant system. Furthermore, it was found that the promoter effect of plant growth is given by a complex regulation of auxin biosynthesis and metabolism, not by an improvement in nutrient uptake.

## Figures and Tables

**Table 1:** Summary of *de novo* assembly statistics of *Eucalyptus globulus* roots by Trinity software.

	*Eucalyptus globulus*
Number of transcripts	131.155
Number of components	81.210
Size	147.15 Mbp
N10	4469 bp
N20	3580 bp
N30	3002 bp
N40	2540 bp
N50	2128 bp
Average length	1121.97 bp
Median	580 bp

**Table 2:** Summary of evaluation of the de novo assembly of *Eucalyptus globulus* roots by BUSCO.

Complete BUSCOs	1133	78.7%
-Single-copy BUSCOs	574	39.9%
-Duplicated BUSCOs	559	38.8%
Fragmented BUSCOs	108	7.5%
Missing BUSCOs	199	13.8%
Total BUSCO genes	1.440	100.0%

**Table 3:** Details of differential expression genes involved in response to heavy metals stress and plant growth promotion in *Eucalyptus globulus* roots transcriptome.

Gene Description	Ontology	FPKM Control	FPKM Treatment	*p*-Value	Up/Down
***Stress responses***					
*Metallothionein-like protein 1*	MF: Metal ion binding (GO:0046872)	69932.98	32933.82	8.65 × 10^−7^	Down
*Peroxidase 10 precursor*	MF: Metal ion binding (GO:0046872), BP: Response to oxidative stress (GO:0006979)	1370.02	615.98	1.61 × 10^−135^	Down
*Probable 2-oxoglutarate-dependent dioxygenase*	BP: Metal ion binding (GO:0046872), Cellular response to toxic substances (GO:0097237)	1049.91	451.091	1.63 × 10^−6^	Down
*Protein Cobra precursor*	BP: Response to salt stress (GO:0006950)	85.18	1617.25	0	Up
*Protein High arsenic content 1, mitochondrial*	BP: Detoxification of arsenic-containing substance (GO:0071722)	1046.34	318.95	5.68 × 10^−34^	Down
*Protein sensitive to proton rhizotoxicity 2*	MF: Metal ion binding (GO:0046872)	234.41	103.58	4.83 × 10^−103^	Down
*Putative Multidrug resistance protein*	MF: ATPase activity, coupled to transmembrane movement of substances (GO:0042626)	161.09	71.71	3.79 × 10^−41^	Down
*Metal tolerance protein 4*	MF: Cation transmembrane transporter activity (GO:0008324)	723.47	280.76	0	Down
*Transcription factor MYB102*	BP: Response to osmotic stress (GO:0006970), response to salt stress (GO:0009651)	120.87	31.99	4.63 × 10^−68^	Down
*Transcription factor MYB74*	BP: Response to salt stress (GO:0009651)	181.21	66	2.09 × 10^−9^	Down
*Transcription factor MYB86*	BP: Regulation of stomatal movement (GO:0010119)	35.69	153.43	3.96 × 10^−23^	Up
*Heat stress transcription factor A-2*	BP: Cellular response to heat (GO:0034605)	298.08	124.75	7.8 × 10^−30^	Down
*Universal stress protein A-like protein*	MF: AMP binding (GO:0016208)	62.21	24.3	2.78 × 10^−10^	Down
*Probable WRKY transcription factor 72*	BP: Defense response (GO:0006952)	344.15	89.65	4.22 × 10^−62^	Down
*Disease resistance protein RPS4B*	BP: Defense response signaling pathway (GO:0009870)	269.97	960.33	0.003	Up
*Disease resistance-like protein DSC1*	MF: Defense response to bacterium (GO:0042742)	269.97	960.33	1.48 × 10^−5^	Up
*Snakin-2 precursor*	BP: Defense response (GO:0006952)	70.25	323.33	5.25 × 10^−21^	Up
*Disease resistance response protein 206*	BP: Defense response (GO:0006952), response to biotic stimulus (GO:0009607)	75.24	308.47	5.89 × 10^−76^	Up
*3,9-dihydroxypterocarpan 6A-monooxygenase*	BP: Defense response (GO:0006952)	234.72	1139.56	1.69 × 10^−25^	Up
*Cysteine protease X CP2 precursor*	MF: Defense response to bacterium (GO:0042742)	403.5	1270.23	0	Up
*Disease resistance protein RPP4*	BP: Defense response (GO:0006952)	239.78	628.32	7.88 × 10^−22^	Up
*Ethylene-responsive transcription factor ERF043*	BP: Ethylene-activated signaling pathway (GO:0009873)	42.47	147.73	1.04 × 10^−40^	Up
***Cell transport***					
*Metal transporter Nramp1*	BP: Iron ion homeostasis (GO:0055072), iron ion transmembrane transport (GO:0034755)	1849.04	706.19	1.68 × 10^−23^	Down
*Metal transporter Nramp3*	BP: Iron ion transmembrane transport (GO:0034755)	1613.53	378.21	3.64 × 10^−34^	Down
*Metal transporter Nramp5*	BP: Iron ion homeostasis (GO:0055072)	1849.04	706.19	0	Down
*Metal transporter Nramp6*	BP: Iron ion transmembrane transport (GO:0034755)	1613.53	378.21	7.81 × 10^–6^	Down
*Heavy metal-associated isoprenylated plant protein 28*	MF: Metal ion binding (GO:0046872), BP: Metal ion transport (GO:0030001)	2010.9	911.05	8.38 × 10^–25^	Down
*High affinity nitrate transporter 2.5*	BP: Cellular response to nitrate (GO:0071249), nitrate transport (GO:0015706)	156.4	53.16	4.63 × 10^−22^	Down
*High-affinity nitrate transporter 3.2*	BP: Nitrate assimilation (GO:0042128), nitrate transport (GO:0015706)	7323.21	3085.33	8.34 × 10^−49^	Down
*Inorganic phosphate transporter 1-1*	BP: Phosphate ion transmembrane transporter activity (GO:0015114)	2858.46	50.92	8.77 × 10^−27^	Down
*Potassium transporter 5*	BP: Potassium ion transport (GO:0006813)	2330.51	570.91	4.86 × 10^−34^	Down
*Sodium transporter HKT1*	BP: Response to osmotic stress (GO:0006970), sodium ion transport (GO:0006814)	116.31	50.95	1.17 × 10^−23^	Down
*Magnesium transporter MRS2-3*	BP: Magnesium ion transport (GO:0015693)	924.34	365.83	6.95 × 10^−139^	Down
*Ammonium transporter 1 member 1*	BP: Ammonium transmembrane transport (GO:0072488)	3059.06	1050.29	0	Down
*High affinity sulfate transporter 1*	MF: Secondary activate sulfate transmembrane transporter activity (GO:0008271)	433.26	100.61	1.59 × 10^−10^	Down
*Aluminum-activated malate transporter 10*	BP: Malate transport (GO:0015743)	379.74	126.8	3.78 × 10^−22^	Down
*Sugar transporter ERD6-like 6*	BP: Glucose homeostasis (GO:0042593)	28.58	102.73	3.24 × 10^−36^	Up
*Probable Aquaporin PIP2-2*	MF: Water channel activity (GO:0015250), BP: Response to water deprivation (GO:0009414)	78.13	1168.64	1.39 × 10^−55^	Up
*Aquaporin TIP1-3*	MF: Water channel activity (GO:0015250), BP: Water transport (GO:0006833)	64.88	252.7	8.49 × 10^−32^	Up
***Plant growth***					
*Auxin-induced protein AUX22*	MF: Auxin-activated signaling pathway (GO:0009734)	106.53	349.04	2.86 × 10^−20^	Up
*Auxin-responsive protein IAA3*	BP: Auxin-activated signaling pathway (GO:0009734), response to auxin (GO:0009733)	18.84	167.76	5.82 × 10^−40^	Up
*Auxin-responsive protein IAA4*	BP: Auxin-activated signaling pathway (GO:0009734)	18.84	167.76	3.26 × 10^−45^	Up
*Auxin-induced protein 22D*	BP: Auxin-activated signaling pathway (GO:0009734)	18.84	167.76	1.33 × 10^−57^	Up
*Auxin efflux carrier component 2*	BP: Auxin-activated signaling pathway (GO:0009734), auxin efflux (GO:0010315)	10.46	115.46	0	Up
*Auxin-induced in root cultures protein 12*	BP: Auxin-activated signaling pathway (GO:0009734)	142.02	772.68	4.19 × 10^−48^	Up
*Auxin-responsive protein SAUR50*	BP: Auxin-activated signaling pathway (GO:0009734), regulation of growth (GO:0040008)	18.44	108.18	1.11 × 10^−15^	Up
*Auxin-responsive protein SAUR78*	BP: Positive regulation of cell growth (GO:0030307), responsive to auxin (GO:0009733)	46.66	107.16	2.42 × 10^−22^	Up
*Auxin- induced protein 22A*	BP: Auxin-activated signaling pathway (GO:0009734)	147.72	441.66	1.15 × 10^−60^	Up
*Indole-3-acetic acid-amino synthetase GH3.17*	BP: Auxin homeostasis (GO:0010252)	41.6	137.52	0	Up
*Transcription factor MYB61*	BP: Response to auxin (GO:0009733), root development (GO:0048364)	35.69	153.43	1.29 × 10^−88^	Up
*Protein RALF-like 24 precursor*	MF: Hormone activity (GO:0005179), BP: Cell-cell signaling (GO:0007267)	182.13	487.29	3.06 × 10^−23^	Up
***Metal-binding***					
*Laccase-15 precursor*	MF: Copper ion binding (GO:0005507), oxidoreductase activity (GO:0016491)	590.37	1885	6.34 × 10^−14^	Up
*Laccase-14 precursor*	MF: Copper ion binding (GO:0005507), oxidoreductase activity (GO:0016491)	675.65	2738.33	9.02 × 10^−51^	Up
*Laccase-16 precursor*	MF: Copper ion binding (GO:0005507), oxidoreductase activity (GO:0016491)	675.65	2738.33	1.01 × 10^−110^	Up
